# Supplementary material for: Splice-Junction-Based Mapping of Alternative Isoforms in the Human Proteome
Source: Cell Rep. Author manuscript; Available in PMC 2020 Jan 15. (PMC6961840; doi:10.1016/j.celrep.2019.11.026)
Supplement: 3 [file NIHMS1546469-supplement-3.zip › DF2/PXD009737/TestisTrypsin-463-Q66K14-LHLPPALSPEEAESALEAAHYFTEDSSSEEALPQEEQEGSGSEER.pdf]

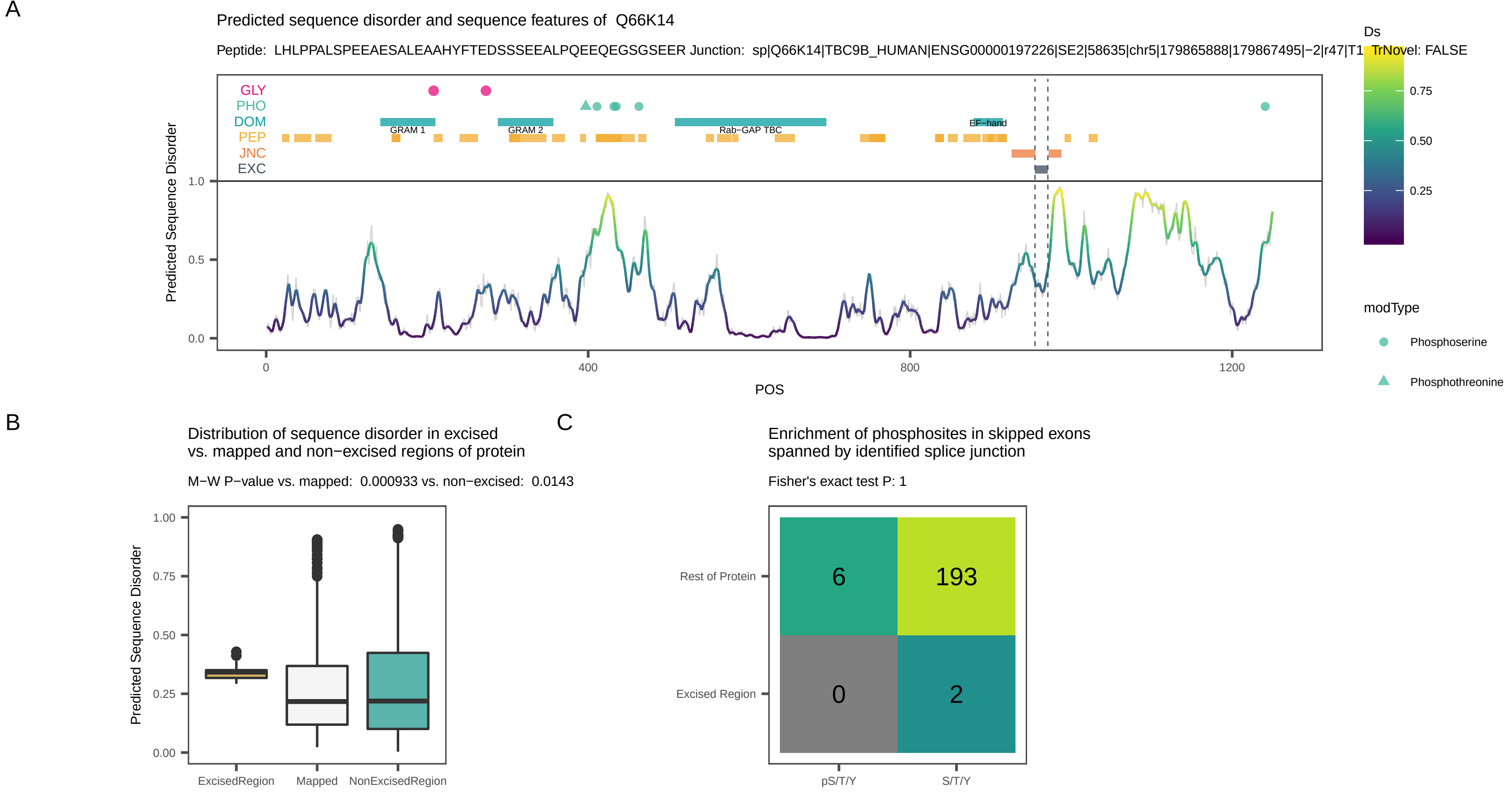
A
Predicted sequence disorder and sequence features of Q66K14
Peptide: LHLPPALSPEEAESALEAAHYFTEDSSSEEALPQEEQEGSGSEER Junction: sp|Q66K14|TBC9B_HUMAN|ENSG00000197226|SE2|58635|chr5|179865888|179867495|−2|r47|T1 TrNovel: FALSE
GLY
PHO
DOM
PEP
JNC
EXC
GRAM 1
GRAM 2
Rab−GAP TBC
EF−hand
Predicted Sequence Disorder
1.0
0.5
0.0
0
400
800
1200
POS
Ds
0.75
0.50
0.25
modType
Phosphoserine
Phosphothreonine
B
Distribution of sequence disorder in excised vs. mapped and non−excised regions of protein
M−W P−value vs. mapped: 0.000933 vs. non−excised: 0.0143
Predicted Sequence Disorder
1.00
0.75
0.50
0.25
0.00
ExcisedRegion
Mapped
NonExcisedRegion
C
Enrichment of phosphosites in skipped exons spanned by identified splice junction
Fisher's exact test P: 1
Rest of Protein
Excised Region
6
193
0
2
pS/T/Y
S/T/Y
